# Large Phenotypic and Genetic Diversity of Prophages Induced from the Fish Pathogen *Vibrio anguillarum*

**DOI:** 10.3390/v11110983

**Published:** 2019-10-24

**Authors:** Daniel Castillo, Nana Andersen, Panos G. Kalatzis, Mathias Middelboe

**Affiliations:** Marine Biological Section, University of Copenhagen, Strandpromenaden 5, DK-3000 Helsingør, Denmark; daniel.castillo@bio.ku.dk (D.C.); nana.b.e.a@hotmail.com (N.A.); panos.kalatzis@bio.ku.dk (P.G.K.)

**Keywords:** Vibrio, prophage, genomics, lysogenic conversion, virulence, aquaculture, H20-like phages

## Abstract

*Vibrio anguillarum* is a marine pathogenic bacterium that causes vibriosis in fish and shellfish. Although prophage-like sequences have been predicted in *V. anguillarum* strains, many are not characterized, and it is not known if they retain the functional capacity to form infectious particles that can infect and lysogenize other bacterial hosts. In this study, the genome sequences of 28 *V. anguillarum* strains revealed 55 different prophage-related elements. Chemical and spontaneous induction allowed a collection of 42 phage isolates, which were classified in seven different groups according to a multiplex PCR assay. One shared prophage sequence, p41 (group III), was present in 17 *V. anguillarum* strains, suggesting that this specific element is very dynamically exchanged among *V. anguillarum* populations. Interestingly, the host range of genetically identical phages was highly dependent on the strains used for proliferation, indicating that phenotypic properties of phages were partly regulated by the host. Finally, experimental evidence displayed that the induced phage ɸVa_90-11-287_p41 was able to lysogenize *V. anguillarum* strain Ba35, and subsequently spontaneously become released from the lysogenized cells, demonstrating an efficient transfer of the phage among *V. anguillarum* strains. Altogether, the results showed large genetic and functional diversity and broad distribution of prophages in *V. anguillarum,* and demonstrated the potential of prophages as drivers of evolution in *V. anguillarum* strains.

## 1. Introduction

Temperate phages can integrate into the host chromosome upon infection. Here, they become prophages and are replicated along with the rest of the bacterial genome when the cell divides [1]. Temperate phages therefore play a major role in the evolution of bacterial communities by transferring genetic information between their bacterial hosts. Genomic sequencing has revealed the presence of prophages in 60–70% of the analyzed bacterial genomes, where they can constitute up to 10–20% of the DNA contributing significantly to intra-species genomic differences [2]. Temperate phages therefore have direct influence on the genetic composition and architecture of the host and potentially enrich the host cell with new beneficial genes (lysogenic conversion), for example by encoding virulence and other fitness factors in pathogenic bacteria [3]. At the same time, prophages represent a potential metabolic burden and molecular time bomb in the host genome, which at any time can reactivate and kill its host [4]. Over longer time-scales, prophages may be domesticated by their hosts, losing their ability to be induced. A number of phage-derived bacterial traits (e.g., toxins and gene transfer agents) originate from conserved prophages, which thus contribute significantly to bacterial evolution [5].

The *Vibrio* genus (vibrios) is a genetically and metabolically diverse group of heterotrophic bacteria that are ubiquitously distributed in the oceans, often accounting for a large fraction (0.5–5%) of the total bacterial community [6,7]. Besides, the group includes several human pathogens (e.g., *V. cholerae* and *V. parahaemolyticus*) [8,9], and pathogens infecting corals (*V. coralliilyticus*) and fish (e.g., *V. anguillarum*, *V. harveyi*) [10,11] and have often been used as model organisms in studies of virulence mechanisms in aquatic systems [12,13]. Despite the ubiquitous distribution of vibrios in the global ocean and their importance as pathogens of humans, fish and marine invertebrates, little is known about the content and role of prophages for the functional properties, pathogenicity and evolution of environmental vibrios. The prophage-encoded toxins CTX and Zot in the human pathogen *V. cholerae* is a classic example of prophage generated virulence in pathogens [14], and the discovery of these prophage-associated toxins in environmental vibrios (e.g., *V. coralliilyticus*, *V. anguillarum*), suggested that prophage-encoded genes are efficiently disseminated between environmental *Vibrio* populations [15]. Besides pathogenicity, prophages have previously been shown to potentially affect fitness and metabolic properties in vibrios. For example, the temperate vibriophage *Vibrio harveyi* myovirus-like (VHML) suppressed metabolic activity in lysogenized *V. harveyi* cells under nutrient-limited conditions [16]. Further, evidence of phage-encoded hemagglutinin, which is potentially involved in virulence of *V. pelagius* [17], and experimental evidence of prophage-mediated virulence in *V. harveyi* [18] support the hypothesis that lysogenic conversion in vibrios represents an important mechanism of adaptation to changing environmental conditions.

In a recent study, the sequencing of 19 *V. anguillarum*-specific temperate bacteriophages isolated across Europe and Chile from aquaculture and environmental sites identified several functional genes, such as N6-adenine methyltransferase and lambda like repressor, as well as the presence of a tRNA^Arg^, which potentially could be beneficial for the host performance [19]. Further, genomic analyses of a collection of 31 virulent *V. anguillarum* strains showed the presence of 19 prophage elements, designated H20-like phages, with low genetic diversity, which were present as prophages in >50% of the strains, covering large geographical distances. The widespread presence of the H20 like-phages suggested that these phages may also represent a significant contribution to the phenotypic properties of *V. anguillarum* upon integration [19]. However, the phenomenon of lysogenic conversion has so far received little attention in non-cholera vibrios, and the occurrence, evolution and exchange of prophage-encoded traits in environmental vibrios are poorly understood [15]. The capacity of phages to encode key functional properties and disseminate these genes among marine bacterial communities emphasizes the need for a better understanding of the role of phage-driven evolution of virulence, fitness factors and efficiency of induction and re-integration of these mobile genetic elements.

In the current study, we aimed at expanding the existing knowledge on the diversity and genomic composition of prophages associated with the fish pathogenic *V. anguillarum*, characterizing the infectious properties of the inducible prophages and examining the potential for dissemination of phage genomes among *V. anguillarum* isolates. Using a collection of 28 whole genome sequenced *V. anguillarum* strains, we demonstrated induction of prophages from half of the strains, representing seven genetically different groups of phages. Spontaneous and chemical induction of phages with highly diverse host range patterns and rapid reintegration in a non-lysogenic strain suggested that prophages are highly dynamic components of the *V. anguillarum* genomes, contributing to a rapid and efficient exchange of genes within this *Vibrio* species.

## 2. Materials and Methods

### 2.1. Identification of Prophages in the Vibrio anguillarum Collection

A collection of 28 whole *V. anguillarum* genomic sequences from aquaculture environments, including strains isolates from rainbow trout, turbot, salmon and sea bass, covering aquaculture facilities from Europe, USA and Chile (distance > 13,000 km, and temporal scales of isolation > 25 years) were analyzed in the study [12,13] (Table S1). The genomic sequences were screened for the presence of prophage-like elements using the web server PHASTER [20]. Selection and characterization of the prophages were based on three specific criteria. Firstly, the genomic similarity of phage-related genes with prophage sequences previously deposited in the PHASTER database. Secondly, the presence of phage-related genes in a DNA sequence should be >50% of the total ORFs (open read frames). Thirdly, the presence of specific phage-related cornerstone proteins (Integrase, fiber, tail, capsid, terminase, protease and lysin), attachment sites, tRNA or short nucleotides repeats should give a score of 10 for each key gene found. Based on these criteria a score value was calculated for each prophage sequence. A prophage-like element was considered incomplete if its completeness score was less than 60, questionable if the score was between 60 and 90, and complete if the score was above 90 [20]. 

In addition, manual identification of prophage-related sequences was done by scanning potential phage-related ORFs using Geneious software v10 [21]. Selected ORFs were compared with known proteins using standard protein-protein BLASTP (August 2018; E-value cutoff <1 × 10^−3^; nonredundant proteins database) at the National Centre for Biotechnology Information (NCBI) [22]. 

### 2.2. Induction and Isolation of Prophages with Mitomycin C

For preparing bacterial strains for prophage induction experiments, strains kept at −80 °C (glycerol 15%) were plated at Marine broth (MB) agar (tryptone 0.5%, yeast extract 0.1%, sea salts 2%, agar 1.5%) and incubated at 28 °C for 24 h. Overnight liquid cultures were prepared from single colonies inoculated in MB broth and incubated for 24 h, 100 rpm, at 22 °C. Induction experiments with mitomycin C were performed for a total of 28 *V. anguillarum* strains (Table S1). Overnight cultures of *V. anguillarum* strains were diluted 1:100 in MB broth and incubated with agitation (100 rpm) at 22 °C for 2.5 h to reach an optical density (OD_600_) ~0.25. Prophage induction was initiated by the addition of mitomycin C (1 μg mL^−1^, final concentration) and OD_600_ was measured every 30 min for 6 h. Two mL aliquots were collected after 6 h of induction and the samples were centrifuged (9000× *g*, 10 min, 4 °C) and the supernatants were 0.22 filtered. Finally, the filtrates were diluted 2× in SM (saline magnesium) buffer (50 mM Tris-HCl, pH 7.5, 99 mM NaCl, 8 mM MgSO_4_, 0.01% gelatin) and stored at 4 °C.

For isolation of induced prophages, the filtrates were spotted on lawns of *V. anguillarum* hosts. The bacterial host strains (Table S1) were grown in 10 mL MB broth at 28 °C for 2.5 h to reach OD_600_ = 0.3. A total of 300 μL of the bacterial strains were added to 4 mL of molten MB top agar (sea salts 1%, agar 0.4%,) and poured on MB agar 1.5% plates. A total of 50 μL of ten-fold serial dilutions in SM buffer of the filtered supernatants were spotted on the lawn of *V. anguillarum* strains and incubated at room temperature for 24 h. Single plaques were isolated directly from the inhibition spots and transferred to 0.5 mL SM buffer, centrifuged (9000× *g*, 10 min, 4 °C) and afterwards stored at 4 °C. The individual plaques were purified 3 times to obtain clonal phage stocks [23]. 

### 2.3. Spontaneous Induction of Prophages

*V. anguillarum* strains, from which phages were induced with mitomycin C, were subsequently examined for spontaneous induction of phages (Table S2). Briefly, single colonies were inoculated in 35 mL of MB broth and incubated at 22 °C, 100 rpm for 12 h. Aliquots of 2 mL (4 and 8 h post inoculation) were centrifuged (9000× *g*, 10 min, 4 °C) and 0.22 μm filtered to allow phage isolation as described above. Individual plaques were purified 3 times to obtain clonal phage stocks [23].

### 2.4. Proliferation of Bacteriophages

A total of 100 µL of bacteriophage stocks were mixed with 300 μL of *V. anguillarum* cells in the exponential growth phase (OD_600_ = 0.3) and incubated at 22 °C for approximately 30 min. A total of 4 mL of 43 °C MB top agar (0.4%) was added, and the mixtures were poured onto a MB agar 1.5% plates, which were immediately placed at 22 °C. After incubation of the plates for 24 h at 22 °C, the presence of lytic bacteriophages in the form of plaques was detected. The bacteriophages were eluted by adding 5 mL of SM buffer on top of the plate and incubated for 2 h with shaking (100 rpm), followed by chloroform fixation (10 μL mL^−1^). Finally, supernatants were centrifuged (9000× *g*, 10 min, 4 °C) and transferred to a new tube. For determination of phage concentrations, serial dilutions in SM buffer were used with the spot assay method [24]. Unless otherwise mentioned, the *V. anguillarum* strains Ba35 and T265 were always used in the double-layer method for proliferation and quantification of phages [23].

### 2.5. Host Range Analysis for Phenotypic Characterization of Induced Phages

A total of 42 phages (26 mitomycin C induced and 16 spontaneously induced) were tested for infectivity against the 32 *V. anguillarum* strains. The host range of the isolated bacteriophages was determined by spotting 10 μL of bacteriophage stocks (10^8^–10^9^ PFU mL^−1^) on top of a MB plates (agar 1.5%) plates prepared with 4 mL of MB top agar (0.4%) inoculated with 0.3 mL of the strains to be tested. The host range was determined with three separate plates for each phage-host combination. The phages were characterized as infective (clear or turbid inhibition zones) or non-infective (no bacteria inhibition). An unweighted-pair group method using average linkages (UPGMA) tree was constructed using the software Treecon, where the sensitivity/no sensitivity matrix was converted to pairwise distances using the Dice similarity coefficient [25].

### 2.6. Bacteriophage Purification by CsCl Density Gradient

Phage purification was done by cesium chloride (CsCl) buoyant density centrifugation described in Castillo et al. [26], but modified to fit the conditions for *V. anguillarum*. Briefly, spontaneous induced bacteriophage ΦVa_90-11-287_p41 was proliferated using the *V. anguillarum* strains Ba35 and T265 as is described previously. Phages stocks (50 mL; 10^9^ PFU mL^–1^) were concentrated by adding poly-ethylene glycol 8000 (PEG-8000) and sodium chloride (final concentration 10% *w*/*v* and 1 M, respectively) and followed by incubation at 4 °C for 24 h. Subsequently, phage solutions were centrifuged (10,000× *g*, 30 min, 4 °C) and the phage pellet was resuspended in 1 mL of SM buffer. Immediately, phage suspensions were centrifuged over a 1.6–1.3 g mL^−1^ CsCl gradient (100,000× *g*, 4 °C, 24 h) in a swinging bucket ultracentrifuge rotor (Beckman SW 55 Ti). Then, the phages were collected from visible bands by puncturing the side of tube with a needle attached to a syringe. The density of the fractions containing the phages was calculated by weighing 100 μL of the fraction. Finally, the concentrated phage solutions were dialyzed against SM buffer by Amicon Ultra-15 Centrifugal Filter Units (Merck Millipore, Berlin, Germany) at 3000× *g*, 4 °C, for 4 h. Phage stocks were quantified by spot assay, diluted to 10^9^ PFU mL^−1^ in SM buffer and stored at 4 °C for subsequent host range analysis.

### 2.7. Linking Induced Phages to Prophages Using PCR Amplification

In order to map the induced phages back to the prophage-like elements detected by PHASTER, primers for a multiplex PCR analyses were designed to target specific phage genes. These regions included phage tail, capsid and hypothetical genes (Table S3).

Phage samples were treated with DNAse and RNAse (both at a final concentration of 1 μg mL^−1^) for 1 h at 37 °C. Inactivation of the enzymes was done at 65 °C for 10 min. The PCR reaction was performed in a 25 μL reaction mixture containing 15.5 μL PCR water, 5 μL PCR 5× red reaction buffer, 0.25 μL BSA 1%, 1 μL Mg^+2^ 25 mM, 0.25 μL DNA polymerase (1.25 units), 0.50 μL of each primer (100 pmol) and 1 μL DNA template (~10^6^ phages mL^−1^). The PCR reaction was run in the thermocycler: 10 min at 96 °C, 30 cycles of 1 min of denaturation at 96 °C, 1 min of annealing at 58 °C, and 1 min of extension at 72 °C, followed by 10 min at 72 °C. PCR products were visualized by agarose 1% gel stained with GelRed Nucleic Acid Stain (Biotium, Fremont, CA, USA). A 100-bp DNA ladder (Omega Bio-Tek, Atlanta, GA, USA) was used as a molecular size marker. 

### 2.8. DNA Extraction, Sequencing and Bioinformatic Analysis

For DNA extraction, 50 mL phage stocks (>10^8^ PFU mL^−1^) were concentrated to 250 μL by centrifugation in Amicon Centrifugal filters (5000× *g*, 25 min, 4 °C, 4 times) and transferred to a clean 1.5 mL tube. Phage samples were treated with DNAse I and RNase A (both a final concentration of 1 μg mL^–1^) at 37 °C for 1 h. Following the incubation, enzymes were subjected to inactivation at 65 °C for 10 min. Immediately, DNA extraction was performed according to Wizard Genomic DNA Purification Kit (Promega, Hilden, Germany). The amount of genomic DNA was measured using Quant-iTTM PicoGreen^®^ quantification kit (Invitrogen, Waltham, MA, USA).

Genome sequencing was conducted using Illumina Hi Seq 2000 sequencer at Beijing Genomic Institute (Shenzhen, Guangdong, China) using pair-end read sizes of 100 bp. Library construction, sequencing, and data pipelining were performed in accordance with the manufacturer’s protocols. The Illumina data-reads were assembled into contiguous sequences by Geneious software version 10 [21], resulting in single contigs for all phages. Screening for potential ORFs and functions of genes was done by ORFinder [21]. Deduced ORF sequences were compared with known proteins using standard protein-protein BLASTP (October 2018; E-value cutoff < 1 × 10^−3^; nonredundant proteins database) at the National Centre for Biotechnology Information (NCBI) [22]. 

To reveal the phylogenetic relationship among *V. anguillarum* phages, the terminase large subunit gene was selected. In addition, terminase sequences from specific vibrio prophage-like elements were also identified by BLASTP (requiring an E-value < 1 × 10^−3^) in our prophage database [15]. For each gene, protein sequences were aligned using ClustalW version 2.0 [27], and phylogeny was inferred using maximum likelihood in Geneious version 10 [21]. Finally, genomic comparison of phage DNA sequences was done using the MAUVE v2.3.1 software [28].

### 2.9. Lysogenization of V. anguillarum Strain BA35 with a Temperate Phage

To examine the ability of temperate phage ΦVa_90-11-287_p41 to integrate into another non-lysogenized *V. anguillarum* strain, a bacteria culture infection experiment was performed. Integration of the temperate phage ΦVa_90-11-287_p41 (isolated from the strain 90-11-287) was examined during exposure to *V. anguillarum* strain Ba35. Triplicate overnight cultures of strain were 100-fold diluted in 50 mL MB broth and phage ΦVa_90-11-287_p41 was added with a MOI of 10. Bacteria cultures were incubated for 12 h, shaking 100 rpm, at 22 °C and every hour OD_600_ was measured to monitor the bacterial growth. Aliquots of 500 μL were collected during the experiment for quantification of phages by spot assay [24]. At the end of the experiment, aliquots from the infected cultures were streaked on MB plates and incubated at 22 °C for 24 h. From these plates, 50 individual colonies were selected and screened for the presence of phage ΦVa_90-11-287_p41 as a prophage in the host genome by a multiplex PCR detection, using primer sets designed for prophage p41 (Table S3). In addition, a spot assay was performed to test whether these clones have developed resistance against the phage ΦVa_90-11-287_p41 and other temperate vibriophages (Table S2). This was verified by spotting 10 μL of phages (10^9^ PFU mL^−1^) onto lawns of the isolates in 4 mL MB top agar (0.4%) on MB plates (1.5%). The inability of the phage to cause cell lysis after 24 h of incubation verified phage resistance in the isolates.

### 2.10. Accession Numbers

The GenBank accession numbers for the sequenced bacteriophages are: bacteriophage ΦVa_90_11_287_p41 (MK672799), bacteriophage ΦVa_90_11_287_p41_Ba35 (MK672800), bacteriophage ΦVa_90_11_287_p41_T265 (MK672801), bacteriophage ΦVa_90-11-286_p16 (MK672802), bacteriophage ΦVa_91-7-154_p41 (MK672803), bacteriophage ΦVa_178/90_p41 (MK672804) and bacteriophage ΦVa_PF430-3_p42 (MK672805).

## 3. Results

### 3.1. In Silico Analysis of Prophage-Like Sequences Pool from V. anguillarum Strains

**Figure 1:**
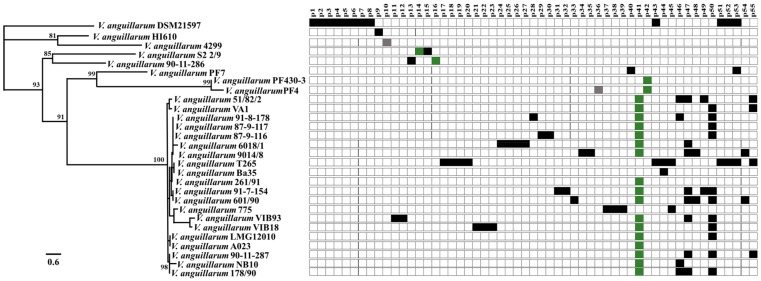
In silico detection and distribution of prophage-like sequences in *V. anguillarum* strains. Unrooted core phylogenetic tree of the 28 *V. anguillarum* strains based on 1723 concatenated genes (both chromosomes) [13]. Bootstrap values of <80% were removed from the tree. The horizontal bar at the base of the figure represents 0.6 substitution per amino acid site. Presence of specific prophage-elements are labeled by colored squares. Green, gray and black squares indicate complete, questionable and incomplete prophage-like elements respectively.

The genomic analysis of 28 *V. anguillarum* strains, representing distinct geographic locations, years of isolation and hosts revealed 55 different prophage-related sequences. In general, prophages were found in both chromosomes and all the *V. anguillarum* strains contained at least one prophage-like element with a maximum 12 prophages in one strain (DSM21597) (Figure 1). Their genomes ranged in size from 5.3 to 53.1 kb, in GC content from 34 to 46.1% and in number of ORFs from 5 to 92. Screening for phage-related genes classified 9% of prophage-like sequences as complete (e.g., p14, p16, p36, p41, p42), 2% questionable (e.g., p10) and 89% as incomplete prophages (e.g., p15 and p33) (Table S4). In addition, 40 out of 55 (72%) of the prophage related-elements were unique for a specific strain. For example, incomplete prophage p40 (19 kb) was unique for *V. anguillarum* strain PF7 (Figure 1; Table S4). The remaining fifteen prophage related elements (28%) were shared among the *V. anguillarum* strains (Table S4). For example, one prophage (p41) was present in 60% of the *V. anguillarum* genomes, covering isolates from Denmark, Greece, Norway, Germany, Finland, Spain, and Italy (Figure 1; Tables S1 and S4). This prophage belonged to the H20-like phages, which is a group of genetically similar temperate phages with a global distribution [19].

Genomic characterization displayed that the prophage-like elements contained a variety of functional genes. Resistance to camphor and fluoroquinolones antibiotics were found in the prophages-like elements p21 and p29, respectively. In addition, toxin related genes such as *zot* (zonula occludens toxin) and *ace* (accessory cholera toxin) were found in the prophage sequences p36 and p44. Similarly, prophage-like element p1 harbored a gene encoding a toxin secretion protein (Table S4). A wide distribution of proteases and peptidases were found in prophages p1, p3, p33, p34 and p43. Interestingly, genes mediating stimulus-response coupling in bacterial chemotaxis (e.g., HipA, chemotaxis, histidine kinase proteins) were found in prophages p10, p28, p31 and p40 (Table S4).

### 3.2. Mitomycin C and Spontaneous Induction of Vibrio anguillarum Prophages

To experimentally confirm the ability of *V. anguillarum* prophages to undergo lysogenic-to-lytic conversion, exponentially growing cells were exposed to mitomycin C (MitC) treatment. From the 28 *V. anguillarum* strains tested for prophage induction, supernatants producing clear plaques on the cell lawns of the *V. anguillarum* strains Ba35 and T65 were obtained from 14 strains. Twenty-eight mitomycin-induced phages were isolated from these strains. The mitomycin-inducible strains were subsequently tested for spontaneous induction of prophages, and in 8 out of the 14 *V. anguillarum* strains spontaneous phage induction occurred, with phage concentrations in the supernatant ranging from 7 × 10^5^ to 1.1 × 10^6^ PFU mL^−1^ during 12 h of bacteria culture incubation. A collection of 14 spontaneously induced phages were isolated using the *V. anguillarum* strains Ba35 and T265 as hosts. All the 42 isolated phages were resistant to chloroform and retained infectivity for >10 days at 4 °C.

### 3.3. Linking Induced Phages to Prophages in Host Genomes

In order to map the induced phages back to prophage sequences in the bacteria genomes, a PCR amplification of prophage-specific genes was performed (Table S3). Results displayed that induced prophages were classified in seven different groups according to multiplex PCR detection (Table 1). For example, 32 out of 42 induced phage isolates belonged to the H20-like prophage, the sequence of which is present in 60% of *V. anguillarum* strains (Table 1) [19] and in this study was identified as p41 in the group III (Table 1; Table S4). Similarly, prophages p16 (group II) and p42 (group V) from the strains 90-11-286 and PF430-3 respectively, were confirmed by PCR analysis. In addition, phages p10 (group I), p44 (group IV) and p40 (group VI) from the strains 4299, Ba35 and PF7 respectively, were classified as the only representatives of these groups (Table 1). Finally, phages isolated from the strain PF7 (group VII) could not be identified by any set of primers designed for that specific strain (Table 1). After linking the phages back to their original prophages, the phage isolates were named according to the original strain and ID number, for example, prophage p41 induced from *V. anguillarum* strain 90-11-287, was named ΦVa_90-11-287_p41.

### 3.4. Phenotypic Characterization of Induced Phages

**Figure 2:**
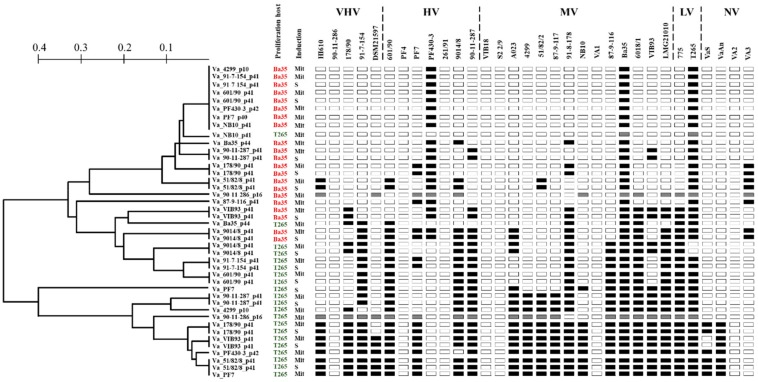
Host range profiles of *V. anguillarum* phages. Phages were grouped based on their infectivity against 32 *V. anguillarum* strains using the unweighted-pair group method. Infectivity is categorized as: white “no inhibition observed”, gray “turbid inhibition zone”, black “clear inhibition zone”. Proliferation host (red or green colors) and induction method (Mit [mitomycin C], S [spontaneous]) were added to facilitate comparison. The virulence ranking of the strains is based on three fish larva models [12]: very high virulence (VHV), high virulence (HV), medium virulence (MV), low virulence (LV) and non-virulence (NV).

The host range analysis of the forty-three purified phages was analyzed by testing infectivity against 32 *V. anguillarum* strains by spot assay. In general, the 42 phages grouped in 22 different phenotypic clusters according to the ability to infect the strains (Figure 2). Together, the phages were able to infect 27 out of 32 strains tested (84%), and only strains 90-11-286, PF4, 261/91, VIB18, S2 2/9, VA1 and VA2 were resistant to all the phages in the collection (Figure 2). Moreover, phages could infect across the collection the *V. anguillarum* strains, independently of host virulence properties obtained from larval challenge experiments [12]. For example, bacteriophage ΦVa_51/82/2_p41 (belonging to the H20-like phages and proliferated in strain Ba35) infected *V. anguillarum* strains HI610, PF430-3, Ba35, T265 and VA3, which represented very high, high, medium, low and non-virulence strains, respectively in a turbot and cod larval system (Figure 2) [12]. Six phages (ΦVa_4299_p10, ΦVa_91-7-154_p41, ΦVa_601/90_p41, ΦVa_NB10_p41, ΦVa_PF430-3_p42 and ΦVa_PF7_p40), proliferated using the strain Ba35, exhibited the narrowest host range, infecting only the strains Ba35, T265 and PF430-3 (Figure 2).

Interestingly, large differences in host susceptibility patterns were observed depending on the proliferation host. In general terms, the host range of the twenty-two phage isolates proliferated using *V. anguillarum* strain BA35 showed a narrow host range, infecting from 9 to 38% of the *V. anguillarum* collection (Figure 2; red color), whereas the same phages proliferated using proliferation strain T265 had a broader host range, infecting from 9 to 72% of the strains (Figure 2; green color). For example, phages ΦVa_51/82/2_p41 and ΦVa_PF7_p40, proliferated using the strain Ba35, only infected 7 (22%) and 3 (9%) of the 32 *V. anguillarum* strains respectively. In comparison, the same phages proliferated using the strain T265, had the broadest host range infecting 23 (72%) out of the 32 *V. anguillarum* strains (Figure 2).

In order to better understand this host range plasticity, we used experimental and in silico approaches to examine potential drivers of the observed differences in host range between identical phages. First, we explored if the presence of specific bacterial co-factors in the lysate may have affected the host range of the phage. Purification of the phages from the two proliferation strains BA35 and T265 using CsCl density gradients was done to reduce potential bacteria-derived co-factors from the lysed proliferation hosts, which may inhibit the growth of other *V. anguillarum* strains. Using bacteriophage Va_90-11-287_p41 as a model, host range analysis of CsCl-purified phages showed to be identical to the host range obtained from the crude lysate, demonstrating that host range expansion was not related to putative antimicrobial agents in the lysed bacteria cultures (Figure S1). Second, phage host ranges may show plasticity in the presence of restriction–modification (R–M) systems in their hosts, as methylation of the phage DNA affects infectivity [29]. Screening for R–M systems into the proliferation host genomic sequences showed that *V. anguillarum* strains Ba35 and T265 harbored identical R–M systems type I and II, and orphan DNA methylases (Table S5). Finally, we determined if these differences in host range patterns of specific phages after proliferation in the *V. anguillarum* strains Ba35 and T265, were associated with changes in the phage genome sequences upon proliferation. Using also bacteriophage ΦVa_90-11-287_p41 as a model, sequencing of phages obtained after proliferation in the strains Ba35 and T265, respectively, showed no genetic differences between phage genomes, indicating that the host range expansion was not associated with genetic changes (Figure S2; see below).

### 3.5. Sequencing of Induced Vibrio anguillarum Phages

**Figure 3:**
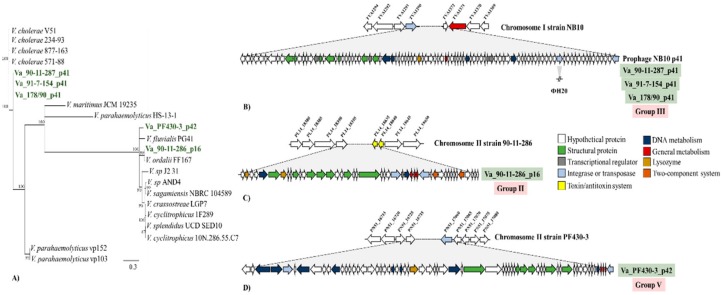
Genetic relationship and schematic representation of *V. anguillarum* temperate phages. (**A**) Phylogenetic relationship of temperate phages based on terminase large subunit protein sequence. To facilitate comparison, terminase amino acid sequences were obtained from *Vibrio* prophage database [15]. Sequenced phages are highlighted in green. (**B**) Genomic organization of phages ΦVa_90-11-287_p41, ΦVa_91-7-154_p41 and ΦVa_178/90_p41, belonging to the H20-like phages (group III). (**C**) Genomic organization of phage ΦVa_90-11-286_p16 (group II). (**D**) Genomic organization of phage ΦVa_PF430-3_p42 (group III). The positions of phages in the bacteria chromosome are specified in the figure and Table S4. The colors were assigned according to the possible role of each ORF as is shown in the figure.

Five phages belonging to group II (ΦVa_90-11-286_p16), group III (ΦVa_90-11-287_p41, ΦVa_91-7-154_p41 and ΦVa_178/90_p41) and group V (ΦVa_PF430-3_p42) were selected for DNA sequencing, phylogeny and identification of possible genomic differences from the original prophage sequences. First, in order to reveal the phylogenetic relationship of these selected phages, the phylogeny was inferred by constructing a terminase-relatedness maximum likelihood tree (Figure 3A). The evolutionary tree displayed three different cluster patterns for all the phages, which varied in the levels of diversity. As expected, all the H20-related phages (group III) grouped in a monophyletic cluster. In contrast, phages ΦVa_90-11-286_p16 and ΦVa_PF430-3_p42 constituted separate clusters (Figure 3A), in which the terminase protein of phage ΦVa_90-11-286_p16 was closely related to a terminase in a prophage-like element in *V. ordalii* strain FF 117 (Figure 3A).

DNA sequences of bacteriophages ΦVa_90-11-287_p41, ΦVa_91-7-154_p41 and ΦVa_178/90_p41 (H20-like phages) revealed that these phages contained 90 ORFs, had a GC content of 43.1% and the presence of an integrase gene. In addition, genomic mapping showed that these phages were localized at the chromosome I in their respective hosts, between transposase (TVA1290) and hypothetical (TVA1372) genes. Genomic comparison at the nucleotide level displayed that phages were 99.9% identical to the prophage sequences from which they originated (Figure 3B). However, a small difference in gene content was found when compared with the previously sequenced phage H20 (Figure 3B) [19], corresponding to the absence of transposase gene in phage H20 (Figure 3B). Similarly, sequencing and annotation of the phage ΦVa_90-11-286_p16 revealed 50 ORFs, a GC content of 44.0% and 100% sequence identity to the prophage sequence in the chromosome II of the *V. anguillarum* strain 90-11-286, located between the genes hypothetical gene (PL14_18395) and toxin/antitoxin system (PL14_18635/18640) (Figure 3C). Finally, annotation of phage ΦVa-PF430-3_p42 showed a 64 ORFs and GC content 44.8%. Subsequently, mapped back to the host PF430-3 revealed also an identical sequence to the respective prophage-sequence located at chromosome II between a hypothetical (PN51_16735) and transposase (PN51_17060) genes (Figure 3D).

### 3.6. Lysogenization of V. anguillarum Strain BA35 with Phage ΦVa_90-11-287_p41

**Figure 4:**
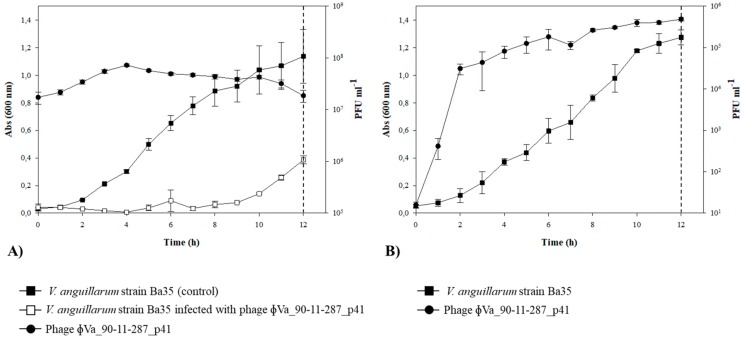
In vitro cell infection experiment of bacteriophage ΦVa_90-11-287_p41 against *V. anguillarum* strain BA35. (**A**) Optical density (OD_600_) of cultures of *V. anguillarum* strain Ba35 amended with phage ΦVa_90-11-287_p41 and control cultures without phage. (**B**) Representative spontaneous release of phage ΦVa_90-11-287_p41 from one lysogenized isolate. PFU mL^−1^ counting was measured every hour. Dotted vertical lines represent time-points of bacterial (**A**) and phage (**B**) isolation. Error bars represent standard deviations from triplicates for each isolate.

In order to evaluate the ability of phages to integrate into the chromosome of potential host bacteria, in vitro infection experiment using the phage ΦVa_90-11-287_p41 and the *V. anguillarum* strain Ba35 was carried out. In the batch culture, the addition of the phages caused a strong control of the density of strain Ba35, relative to the control culture, followed by a re-growth of bacterial cells after 10 h of incubation. Phage abundance increased one order of magnitude over the experiment (Figure 4A). Fifty phage-tolerant clones were selected 12 h post infection and tested for phage susceptibility. Interestingly, all the selected clones were resistant to entire collection of 54 phages, including the lytic broad host range phage KVP40 (Table S2). In addition, screening of lysogenic cells by a multiplex PCR showed that phage ΦVa_90-11-287_p41 was successfully integrated into the bacteria chromosome in all the phage-resistant clones.

To examine spontaneous release of the phage ΦVa_90-11-287_p41 from the lysogenized cells during bacterial growth, the phage production was quantified in three independent lysogenized isolates during 12 h of incubation, using the strain Ba35 as indictor host (Figure 4B). The results of one representative lysogenized clone displayed that the isolates released phages during incubation (Figure 4B). The concentration of phage increased from 10^1^ to ~5 × 10^5^ PFU mL^−1^ after 8 h of incubation. Single plaques isolation (50 plaques) and multiplex PCR assay confirmed the specific release of the phage ΦVa_90-11-287_p41.

## 4. Discussion

The 55 *V. anguillarum* prophage-related sequences identified in the present study represented a large diversity and the contribution to the genetic diversity of *V. anguillarum* strains (Figure 1; Table S4), supporting previous observations that mobile genetic elements (MGE) are major driving forces in the evolution of *V. anguillarum* [13]. In addition to the genetic diversity of the prophages, large differences in host range patterns between genomically identical phages were proliferated in two different *V. anguillarum* hosts (strains T265 and BA35), suggested that phage host range was affected by host-specific, non-genomic mechanisms, which affected phage functional properties, thus expanding phage diversity beyond genetic variation (Figure 2). Exploring the complex composition, dynamics and distribution of *V. anguillarum*-specific prophages thus provided new insights in bacteriophage biology.

The presence of inducible prophages in *Vibrio* species has previously been shown in several studies [30,31,32,33], and it was therefore expected that a part of the 55 prophages in the *V. anguillarum* strains were inducible (Table 1). The chemical induction on *V. anguillarum* strains by mitomycin C from 14 *V. anguillarum* strains (Table 1) agrees with an early study where prophages from *V. campbellii* were successfully induced by mitomycin at the same concentration (1 μg/mL) [30]. Interestingly, 36% of the isolated *V. anguillarum* phages were induced spontaneously from the bacteria cultures (Table 1; Figure 2). Similar findings have been reported for *V. cholerae* [34] and also for other bacteria such as *Shewanella oneidensis* [35], *Corynebacterium glutamicum* [36] and *Flavobacterium psychrophilum* [37]. It has been speculated that spontaneous induction of phages may be a host-driven mechanism for outcompeting closely related strains via viral lysis, giving them a competitive advantage against non-lysogenized cells, while they are at the same time immune to re-infection by the phage (superinfection exclusion) [38,39,40,41]. However, the infection abilities of spontaneously induced *V. anguillarum* phages showed highly variable patterns in the current study. First, the host range analysis displayed that 50% of these phages were able to re-infect their prophage-carrying host (Figure 2). For example, phages ΦVa_90-11-287_p41, ΦVa_51/82/2_p41 and ΦVa_VIB93_p41 (all belonging to the group III) had the capacity to lyse their own particular host, and for other phages, this ability appeared when another host was used a proliferation host (Figure 2). Consequently, prophage-generated immunity to induced phages was not the general pattern in *V. anguillarum*. This finding is line with previous studies in the fish pathogen *F. psychrophilum*, where 6H prophage-carrying strain was at the same time the proliferation host for the phage [42]. Second, when the *V. anguillarum* strain Ba35 was infected with the phage ΦVa_90-11-287_p41, the lysogenized clones were indeed resistant to the induced phage, suggesting that in some cases, lysogeny may provide resistance against re-infection. In these experiments, however, other resistance mechanisms may play a role, due to the strong lytic control of the ΦVa_90-11-287_p41 (Figure 4A). Generally, our results showed no clear patterns of prophage-mediated protection against similar phages, as is generally assumed [43], emphasizing the complexity of phage-defense mechanisms in *V. anguillarum* [44]. This is also in line with the recent demonstration of a prophage-mediate phage defense system in *Mycobacterium*, which was shown to be highly phage specific and depending on the expression of specific prophage-encode genes [45].

The unexpected host range plasticity of *V. anguillarum* phages observed here, contrasted with the narrow host ranges often found in temperate *Vibrio* phages [19,46]. The strong host-range dependence on proliferation hosts, with much broader host ranges of phages produced by *V. anguillarum* strain T265 than strain Ba35 (Figure 2), suggested a host-controlled variation in phage-host range. Interestingly, the differences in host range were not caused by microbial components from the lysed proliferation hosts (Figure S1) or genetic mutations after proliferation in the *V. anguillarum* strains Ba25 and T265, respectively (Figure S2). This phenomenon of host-induced modification of phages, first described by Luria and Human (1952) [47], has been ascribed to specific methylation patterns of the host R–M systems, which are passed on to the progeny phages that evade the R–M system of these hosts [48]. Specific methylation patterns modify specific target sequences in the phage, which will therefore become invisible to that restriction system during subsequent infections [29,49]. However, analysis of genes related to R–M systems in both *V. anguillarum* strains did not reveal any genetic differences between the hosts (Table S5). The H20-like phages (Group III- phages, Table 1) have previously been shown to encode its own methyl transferase gene (locus tag: H20_0036) [19], which may provide protection against host restriction enzymes [50]. The phages may therefore also influence their own host range, if the phage-driven methylation varies between different hosts of proliferation. We suggest that the variability in phage host range in identical *V. anguillarum* phages is attributed to methylation of phage DNA during proliferation. However, more studies are required to resolve the specific mechanisms behind the phage host range variations.

Prophages can modify the lifestyle, fitness, virulence, and evolution of their bacterial host in numerous ways [51], and the current analysis supports that prophages in *V. anguillarum* contribute with important virulence and other fitness factors among the 23% prophages encoding putative functions (Table S4). While the majority (90%) of the prophages are incomplete and likely have become a permanent part of the host genome, the inducible prophages showed to be a dynamic component of the *V. anguillarum* community with a strong potential for dispersal within the population. This was particularly demonstrated by the H20-like phages (genome group III), which were found in >60% of the isolates and showed a global distribution [19]. The current mapping and characterization of prophage elements in *V. anguillarum* is thus a starting point for exploring in more detail the role of temperate phages for pathogenicity and other functional properties in this important pathogen.

The 55 prophage-like sequences in 28 available *V. anguillarum* genomic sequences represent a minimum estimate of the prophage diversity, as we cannot rule out the presence of unrecognized prophages in our *V. anguillarum* collection. This is partly due to the large number of phage-ORFs which are not related to known phage genes in databases, and partly due to limited number of potential sensitive indicator host for detection of inducible phages [52]. Similar observations have been described in the marine bacterium *Roseovarius nubinhibens*, where no prophage sequences were detected by bioinformatic analysis, whereas chemical induction led to the identification and characterization of an unknown *Siphoviridae* phage [53]. The Group VII-phage Va_PF7 (Table 1) is an example of such a hidden prophage which was not recognizable by PHASTER, but appeared in the lysate.

The use of lytic vibriophages against fish pathogens in aquaculture has demonstrated great potential to control pathogens and decrease fish mortality [44,54,55]. As temperate phages can enter either lytic or lysogenic lifecycle, strictly lytic phages are preferred over temperate phages in phage therapy context [55]. Despite the many concerns about the therapeutic use of temperate phages, these phages also possess particularly interesting advantageous features. Currently, temperate phages have been taken under consideration to engineer delivering synthetic gene networks which interfere with bacterial metabolic process with the aim of causing bacterial death [56] or resensitizing the bacteria to antimicrobials [57]. In addition, genetic deletion of genes involved in the lysogenic cycle could be a strategy to drive temperate phages to a strict lytic cycle [58]. Based on our results of the distribution of prophage-related sequences (Figure 1), host range expansion (Figure 2), genetic organization (Figure 3) and the high frequency of integration (Figure 4), *V. anguillarum* temperate phages might be an excellent model to explore mutant temperate phages for therapeutic purposes and thus establishing new foundations for the successful and safe control of this marine pathogen in aquaculture environments.

## 5. Conclusions

The detection of 55 different prophage-like elements in 28 *V. anguillarum* strains suggested that prophages likely play an important role in the biology, gene exchange and evolution of this marine pathogenic bacterium. Interestingly, high rates of spontaneous induction and re-integration were observed in a widely distributed group of H20-like prophages, with potentially strong effects on bacterial competition, population dynamics and functional properties. In addition, large differences in host pattern were observed when identical phages were proliferated using two different *V. anguillarum* strains (T265 and BA35), indicating that this host range expansion is partially regulated by the host. Thus, occurrence, diversity and dynamics of *V. anguillarum* prophages provide insight into the potential of these elements as drivers of gene exchange in *V. anguillarum* communities.

## Figures and Tables

**Table 1:** Group of induced phages from *V. anguillarum* strains.

Group	Phage	Prophage Host	Induction Type	Genome Size kb (Prophage)	Proliferation Host (s)
I	Va_4299_p10	4299	Mitomycin C	9.7	T265; Ba35
II	Va_91-11-286_p16	90-11-286	Mitomycin C	41.2	T265; Ba35
III	Va_VIB93_p41	VIB93	Mitomycin C; spontaneous	53.1	T265; Ba35
	Va_90-11-287_p41	91-11-287	Mitomycin C; spontaneous	53.1	T265; Ba35
	Va_51-82-2_p41	51-82-2	Mitomycin C; spontaneous	53.1	T265; Ba35
	Va_87-9-116_p41	87-9-116	Spontaneous	53.1	Ba35
	Va_91-7-154_p41	91-7-154	Mitomycin C; spontaneous	53.1	T265; Ba35
	Va_601/90_p41	601/90	Mitomycin C; spontaneous	53.1	T265; Ba35
	Va_178/90_p41	178/90	Mitomycin C; spontaneous	53.1	T265; Ba35
	Va_9014/8_p41	9014/8	Mitomycin C; spontaneous	53.1	T265; Ba35
	Va_NB10_p41	NB10	Mitomycin C	53.1	T265; Ba35
IV	Va_BA35_p44	Ba35	Mitomycin C	9.2	T265; Ba35
V	Va_PF430-3_p42	PF430-3	Mitomycin C	52.9	T265; Ba35
VI	Va_PF7_p40	PF7	Mitomycin C	19.0	T265; Ba35
VII	Va_PF7	PF7	Spontaneous	ND	T265

## Supplementary Materials

The following are available online at https://www.mdpi.com/1999-4915/11/11/983/s1, Table S1: Vibrio anguillarum strains used in this study, Table S2: Vibrio anguillarum-specific phages used in this study, Table S3: Sequence of the PCR primers used in this study, Table S4: Prophage-like elements in a collection of 28 Vibrio anguillarum strains. Table S5: Restriction–Modification systems in V. anguillarum strains, Figure S1: Host range analysis of purified bacteriophage Va_90-11-287_p41, Figure S2: Genomic comparison of bacteriophage ɸVa-90-11-287_p41 proliferated in the V. anguillarum strains Ba35 and T265.
