# Supplementary material for: Large Phenotypic and Genetic Diversity of Prophages Induced from the Fish Pathogen Vibrio anguillarum
Source: Viruses. 2019 Oct 24;11(11):983. doi: 10.3390/v11110983 (PMC6893619; doi:10.3390/v11110983)
Supplement: Supplementary file 1 [file viruses-11-00983-s001.pdf]

Supplementary information

Table S1. *Vibrio anguillarum* strains used in this study cited.

Strain	Origin	Year isolation	Fish host	Accession numbers	Reference
4299	Norway	Unknown	Unknown	CP011458/CP011459	[13]
87-9-116	Finland	1987	Atlantic salmon	CP010044/CP010045	[13]
87-9-117	Finland	1987	Rainbow trout	CP010046/CP010047	[13]
90-11-286	Denmark	1990	Rainbow trout	CP011460/CP011461	[13]
90-11-287	Denmark	1990	Rainbow trout	CP011475/CP011476	[13]
91-7-154	Denmark	1991	Turbot	CP010082/CP010083	[13]
178/90	Italy	Unknown	Sea bass	CP011470/CP011471	[13]
601/90	Italy	Unknown	Sea bass	CP010076/CP010077	[13]
775	US	Unknown	Coho salmon	CP002284/CP002285	[13]
9014/8	Denmark	1990	Rainbow trout	CP010038/CP010039	[13]
DMS21597	Norway	Unknown	Atlantic cod	CP010084/CP010085	[13]
HI610	Norway	Unknown	Atlantic cod	CP011462/CP011463	[13]
NB10	Sweden	Unknown	Unknown	LK021130/LK021129	[13]
PF4	Chile	2004	Salmon salar	CP010080/CP010081	[13]
PF7	Chile	2004	Salmon salar	CP011464/CP011465	[13]
PF430-3	Chile	2013	Unknown	CP011466/CP011467	[13]
S2 2/9	Denmark	Unknown	Rainbow trout	CP011472/CP011473	[13]
VA1	Greece	2014	Sea bass	CP010078/CP010079	[13]
6018/1	Denmark	Unknown	Rainbow trout	CP010291/CP010292	[13]
VIB18	Denmark	Unknown	Rainbow trout	CP011436/CP011437	[13]
261/91	Italy	Unknown	Sea bass	CP010032/CP010033	[13]
A023	Spain	Unknown	Turbot	CP010036/CP010037	[13]
LMG12010	Unknown	Unknown	Unknown	CP011468/CP011469	[13]
T265	UK	Unknown	Atlantic salmon	CP010040/CP010041	[13]
51/82/2	Germany	Unknown	Rainbow trout	CP010042/CP010043	[13]
VIB93	Denmark	1985	Rainbow trout	CP011438/CP011439	[13]
91-8-178	Norway	1991	Turbot	CP010034/CP010035	[13]
Ba35	US	Unknown	Sockeye salmon	CP010030/CP010031	[13]
VaS	Greece	2012	Sea bass	NA	[59]
VaAn	Greece	Unknown	Mussel	NA	[59]
VA2	Denmark	2014	Atlantic salmon	NA	[59]
VA3	Denmark	2014	Atlantic salmon	NA	[59]

NA: not applicable.

Table S2. *Vibrio anguillarum*-specific phages used in this study.

Phage	Origin	Method isolation	Host isolation	Host proliferation	Accession number	Reference
KVP40	Japan	W	PF430-3	PF430-3	NC_005083	[44]
ϕH2	Denmark	W	A023	Ba35	KY658673	[19]
ϕH8	Denmark	W	T265	Ba35	KY658674	[19]
ϕH20	Denmark	W	Ba35	Ba35	KY658675	[19]
ϕP2	Norway	W	Ba35	Ba35	KY658676	[19]
ϕP3	Norway	W	Ba35	Ba35	KY658677	[19]
ϕpVa-1	Greece	W	Ba35	Ba35	KX581095	[19]
ϕpVa-2	Greece	W	Ba35	Ba35	KX581094	[19]
ϕpVa-3	Greece	W	Ba35	Ba35	KY658678	[19]
ϕpVa-4	Greece	W	Ba35	Ba35	KY658679	[19]
ϕpVa-5	Greece	W	Ba35	Ba35	KX581096	[19]
ϕpVa-6	Greece	W	Ba35	Ba35	KX581097	[19]
ϕpVa-7	Greece	W	Ba35	Ba35	KX581110	[19]
ϕpVa-8	Greece	W	Ba35	Ba35	KY658680	[19]

ϕCLA	Chile	W	Ba35	Ba35	KX581091	[19]
ϕHer	Greece	W	Ba35	Ba35	KX581090	[19]
ϕLen	Greece	W	Ba35	Ba35	KX581092	[19]
ϕPel	Greece	W	Ba35	Ba35	KX581093	[19]
ϕStrym	Greece	W	Ba35	Ba35	KX581099	[19]
ϕVaK	Greece	W	Ba35	Ba35	KX581098	[19]
Va_4299_p10	Denmark	MI	4299	Ba35/T265	NA	This study
Va_91-11-286_p16	Denmark	MI	90-11-286	Ba35/T265	MK672802	This study
Va_VIB93_p41	Denmark	MI	VIB93	Ba35/T265	NA	This study
Va_VIB93_p41	Denmark	SI	VIB93	Ba35/T265	NA	This study
Va_90-11-287_p41	Denmark	MI	90-11-287	Ba35/T265	MK672799	This study
Va_90-11-287_p41	Denmark	SI	90-11-287	Ba35/T265	NA	This study
Va_51-82-2_p41	Denmark	MI	51-82-2	Ba35/T265	NA	This study
Va_51-82-2_p41	Denmark	SI	51-82-2	Ba35/T265	NA	This study
Va_87-9-116_p41	Denmark	SI	87-9-116	Ba35	NA	This study
Va_91-7-154_p41	Denmark	MI	91-7-154	Ba35/T265	MK672803	This study
Va_91-7-154_p41	Denmark	SI	91-7-154	Ba35/T265	NA	This study
Va_601/90_p41	Denmark	MI	601/90	Ba35/T265	NA	This study
Va_601/90_p41	Denmark	SI	601/90	Ba35/T265	NA	This study
Va_178/90_p41	Denmark	MI	178/90	Ba35/T265	MK672804	This study
Va_178/90_p41	Denmark	SI	178/90	Ba35/T265	NA	This study
Va_9014/8_p41	Denmark	MI	9014/8	Ba35/T265	NA	This study
Va_9014/8_p41	Denmark	SI	9014/8	Ba35/T265	NA	This study
Va_NB10_p41	Denmark	MI	NB10	Ba35/T265	NA	This study
Va_BA35_p44	Denmark	MI	Ba35	Ba35/T265	NA	This study
Va_PF430-3_p42	Denmark	MI	PF430-3	Ba35/T265	NA	This study
Va_PF7_p40	Denmark	MI	PF7	Ba35/T265	NA	This study
Va_PF7	Denmark	SI	PF7	T265	NA	This study

W: enrichment water sample; SI: spontaneous induction; MI; mitomycin induction; NA: not applicable.

Table S3. Sequence of the PCR primers used in this study.

Prophage	Status	Target primers	Nucleotide sequence	Amplicon (bp)
p10	Questionable	Hypothetical protein	TCGCAATACGTTCAAGTGCA (forward) TCCCACACACCGTTATAGGC (reverse)	385
		Transporter protein	CCGCCTTTCTTGGATTTGCC (forward) GGCTCTGTCTCGTTGACCAA (reverse)	502
p16	Complete	Hypothetical protein	AGAATACGTCGCCGACCATC (forward) CACTTCTGGCCAAGGGTGAT (reverse)	367
		Phage tail tape measure protein	GCGATAAATTGCGCGGTCTT (forward) CGCGCCATCACGTTAGAAAG (reverse)	646
p40	Questionable	DNA primase	TGCTTGCTGCCCATTCCATA (forward) CTGACCGAAGTTTTTGCGCA (reverse)	427
		carboxylate synthase	GCGGACGAGGCATATCTTCA (forward) TGCGCGCGTGAAATCATTAG (reverse)	350
p41	Complete	Phage capsid protein	CGAAGAAACTCGCGCATCAG (forward) GTGCGGCGTTGTTGGTAAAT (reverse)	389
		Terminase	TACCACGAACACGAAAGCGA (forward) GATAGTCAACCTCGGCTCCG (reverse)	673
p42	Complete	Hypothetical protein	AATTTCCTGCACTCGCTCGA (forward) AAAGCCCTGATTTCTGGCGA (reverse)	396
		Phage tail tape measure protein	ACACGCTAGATAAGAGCCGC (forward) GTCACCCAAAAACGAACCGG (reverse)	608
p44	Incomplete	Zot protein	GTTCCGTCTGGTGTTTTGGC (forward) GCGCTCTCAATGTGTTAGCG (reverse)	432
		Replication initiation protein	AAGTGTCGGGCAAATCGAGT (forward) TCGTTGCTCTGGGTGGTAAC (reverse)	675

Table S4. Prophage-like elements in a collection of 28 *Vibrio anguillarum* strains.

Prophage ID	Host	Size[a] (kb)	N° ORFs	GC %	Gene loci (chromosome)	Feature(s)	Status

p1	DSM21597	11.6	12	45.4	PL85_11475-PL85_11535 (CI)	Protease (S8), hydrolases, DNA metabolism	Incomplete
p2	DSM21597	11.2	11	44.4	PL85_13375-PL85_18205 (CII)	ABC-transporter	Incomplete
p3	DSM21597	8.9	9	44.4	PL85_10610-PL85_10660 (CI)	Aminopeptidase (*pepB*), cysteine desulfurase (*IscS*)	Incomplete
p4	DSM21597	8.7	9	45.0	PL85_14245-PL85_17570 (CII)	Unknown function proteins	Incomplete
p5	DSM21597	7.4	7	43.0	PL85_13205-PL85_13225 (CI)	Unknown function proteins	Incomplete
p6	DSM21597	7.2	7	37.9	PL85_17930-PL85_13695 (CI)	Unknown function proteins	Incomplete
p7	DSM21597	6.5	6	42.7	PL85_13580-PL85_11700 (CII)	Unknown function proteins	Incomplete
p8	DSM21597	5.5	5	45.0	PL85_12645-PL85_12675(CI)	MBL fold hydrolase	Incomplete
p9	HI610	6.7	7	42.2	PO29_18085-PO29_14265 (CII)	Phage-related proteins	Incomplete
p10	4299	9.7	10	42.5	AA407_17005-AA407_17060 (CII)	Chemotaxis protein	Questionable
p11	VIB93	20.7	20	41.4	AA406_14555-AA406_14655 (CII)	Toxin secretion protein, ABC transporter	Incomplete
p12	VIB93	16.5	27	41.9	AA406_17655-AA406_17780 (CII)	Phage N-6-adenine-methyltransferase, BAX inhibitor protein	Incomplete
p13	90-11-287	16.1	19	45.9	QR76_17890-QR76_17985 (CII)	Phage-related proteins	Incomplete
p14	S2 2/9	44.3	41	43.3	PO25_10445-PO25_16925 (CII)	Phage-related proteins	Complete
p15	S2 2/9	22.8	10	45.6	PO25_17350-PO25_17395 (CII)	ABC transporters	Incomplete
p16	90-11-286	41.2	42	44.0	PL14_18255-PL14_18545 (CII)	Phage-related proteins	Complete
p17	T265	9.6	11	34.0	PN44_01820-PN44_01865 (CI)	Unknown function	Incomplete
p18	T265	8.4	9	45.7	PN44_01865-PN44_01865 (CI)	DNA metabolism	Incomplete
p19	T265	8.4	9	45.0	PN44_17430-PN44_17430 (CII)	Phage-related proteins	Incomplete
p20	T265	5.3	6	40.4	PN44_17515-PN44_17550 (CII)	Transcriptional regulator XRE, unknown function	Incomplete

p21	VIB18	28.9	33	43.3	AA405_17355-AA405_17530 (II)	Lipocalin protein, camphor resistance protein (*crcB*)	Incomplete
p22	VIB18	27.6	40	43.6	AA405_17580-AA405_17850 (CII)	Unknown function proteins	Incomplete
p23	VIB18	10.1	10	44.4	AA405_17535-AA405_17535 (CII)	Unknown function proteins	Incomplete
p24	6018/1	28.7	30	40.7	PN38_17825-PN38_17980	Unknown function proteins	Incomplete
p25	6018/1	22.6	23	44.4	PN38_17600-PN38_17705 (CII)	Unknown function proteins, transporters	Incomplete
p26	6018/1	18.9	17	44.8	PN38_14115-PN38_14205 (CII)	Phage-related proteins	Incomplete
p27	6018/1	18.4	22	42.7	PN38_17710-PN38_17820 (CII)	Phage N-6-adenine-methyltransferase, unknown function proteins	Incomplete
p28	91-8-178	26.6	31	41.7	PN47_17655-PN47_17655 (CII)	Histidine kinase, unknown function proteins	Incomplete
p29	87-9-116	49.2	52	41.9	PO30_17830-PO30_18175 (CII)	Antibiotic resistance protein (*marC*), toxin-antitoxin system	Incomplete
p30	87-9-116	9.5	10	47.0	PO30_17680-PO30_17680 (CII)	Fe^{3+}-hydroxamate ABC transporter permease (*fhuB*)	Incomplete
p31	91-7-154	20.4	26	47.6	PL84_17910-PL84_18045 (CII)	Chemotaxis protein, DNA methylase	Incomplete
p32	91-7-154	20.0	25	46.4	PL84_17765-PL84_17905 (CII)	Unknown function proteins	Incomplete
p33	601/90	31.6	37	45.1	PL15_18175-PL15_18375 (CII)	Metalloprotease (*pmbA*)	Incomplete
p34	9014/8	27.9	27	42.2	PO28_01635-PO28_01667 (CI)	Zinc protease	Incomplete
p35	9014/8	28.2	40	41.7	PO28_13530-PO28_17360 (CII)	Mu-like prophage FluMu F protein	Incomplete
p36	PF4	9.8	15	40.2	CKX99_04400-CKX99_04475 (CII)	Zot and Ace proteins. Filamentous phage	Complete
p37	775	30.5	29	44.6	VAA_03062-VAA_03034 (CI)	Pullulanase	Incomplete
p38	775	19.0	20	41.1	VAA_00599-VAA_02400 (CI)	Unknown function proteins	Incomplete
p39	775	7.2	9	43.1	VAA_00673-VAA_00015 (CI)	Unknown function proteins	Incomplete
p40	PF7	19.0	21	42.7	AA909_11340-AA909_11435 (CI)	HipA protein	Incomplete

p41	NB10*; 87-9-116; 87-9-117; 90-11-287; 91-7-154; 178/90; 601/90; 9014/8; VA1; 6018/1; VIB18; 261/91; A023; LMG12010; 51/82/2; VIB93; 91-8-178	53.1	92	43.4	VANGNB10_cI1581c-VANGNB10_cI1654c (CI)	H20-like prophage	Complete
p42	PF4*; PF430-3	52.9	63	44.8	CKX99_02080-CKX99_02400 (CII)	Threonine-tRNA ligase, unknown function proteins	Complete
p43	DSM21597*; T265	8.1	7	43.9	PL85_15785-PL85_15755 (CII)	Diguanylate cyclase, peptidase	Incomplete
p44	Ba35*; T265	9.2	10	42.9	PN48_17420-PN48_17465 (CII)	Zot and Ace proteins.	Incomplete
p45	775*; T265	9.1	10	39.6	VAA_02475-VAA_02483 (CI)	Filamentous phage O-antigen export system	Incomplete
p46	91-8-178*; NB10; 178/90; 51/82/2	12.5	21	41.9	PN47_17438-PN47_17460 (CII)	Unknown function proteins	Incomplete
p47	VIB93*; 51/82/2; 178/90; 9014/8; 6018/1; 91-7-154; 90-11-287; 601/90	17.9	21	49.8	AA406_17550-AA406_17650 (CII)	Phage-related proteins	Incomplete
p48	601/90*; 9014/8	19.2	19	41.4	PL15_17835-PL15_17935 (CII)	Phage-related proteins	Incomplete
p49	51/82/2*; 91-7-154	24.4	26	41.8	PN46_17530-PN46_17650 (CII)	Phage-related proteins	Incomplete
p50	LMG12010*;90-11-287; 91-7-154; VIB93; 87-9-116; 87-9-117; 91-8-178; 178/90; VIB18; VA1;601/90	10.8	20	41.4	PN43_17070-PN43_17165 (CII)	Phage-related proteins	Incomplete
p51	DSM21597*; T265	10.4	7	44.6	PL85_10800-PL85_10830 (CI)	c-di-GMP phosphodiesterase	Incomplete

p52	DSM21597 *; T265	8.9	11	46.1	PL85_10610- PL85_10660 (CI)	Peptidase (*pepB*)	Incomplete
p53	T265*; DSM21597; PF7	8.4	10	45.7	PN44_12100- PN44_12060 (CI)	Phage-related proteins	Incomplete
p54	9014/8*; 601/90	19.2	20	41.4	PO28_14555- PO28_14650 (CI)	Phage-related proteins	Incomplete
p55	51/82/2*; T265; 90-11-287; VA1	18.5	23	43.4	PN46_17885- PN46_17990 (CII)	Unknown function proteins	Incomplete

*used as reference for genomic localization; [a]: according to the PHASTER prediction.

Table S5. Restriction-Modification systems in *V. anguillarum* strains.

Chromosome	Strain(s)	Function	Locus tag
I	Ba35/T265	Restriction endonuclease	PN48_03160/ PN44_03160
	Ba35/T265	DNA (cytosine-5-)-methyltransferase CDS	PN48_03165/ PN44_03165
	Ba35/T265	Restriction endonuclease	PN48_06310/ PN44_06290
	Ba35/T265	Restriction endonuclease subunit S	PN48_10625/ PN44_10590
	Ba35/T265	Restriction endonuclease subunit R	PN48_10630/ PN44_10595
	Ba35/T265	Restriction endonuclease	PN48_10635/ PN44_10600
	Ba35/T265	Restriction endonuclease subunit M	PN48_12250/ PN44_12195
	Ba35/T265	DNA adenine methylase	PN48_02505/ PN44_02505
	Ba35/T265	DNA methylase N-4	PN48_12230/ PN44_12175

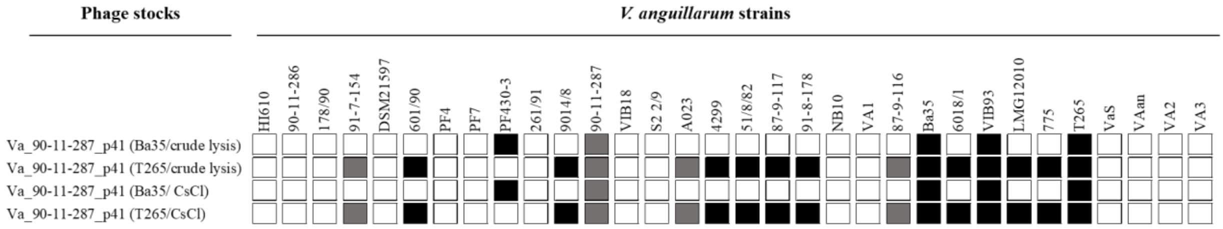

Figure S1. Host range analysis of purified bacteriophage Va_90-11-287_p41. Infectivity is categorized as: white "no inhibition observed", gray "turbid inhibition zone", black "clear inhibition zone". Proliferation host and method of phage purification are indicated in parenthesis respectively.

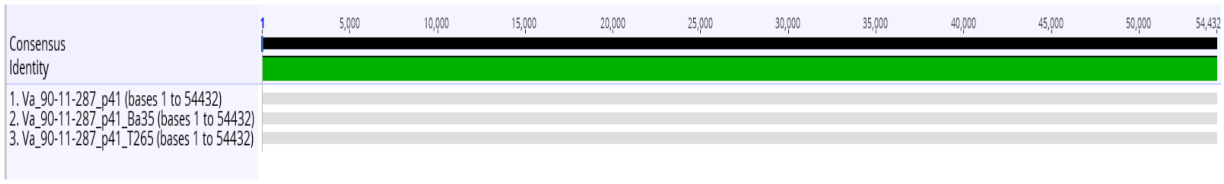

Figure S2. Genomic comparison of bacteriophage ϕVa-90-11-287_p41 proliferated in the *V. anguillarum* strains Ba35 and T265. Grey bars represent the phage genomic sequence with 100% homology among phages. Green color in the consensus sequence indicates identical nucleotide sequence at the same position.

Tan, D.; Gram, L.; Middelboe, M. Vibriophages and Their Interactions with the Fish Pathogen *Vibrio anguillarum*. *Appl. Environ. Microbiol.* **2014**, *80*, 3128–3140.
